# Role of p16/MTS1, cyclin D1 and RB in primary oral cancer and oral cancer cell lines

**DOI:** 10.1038/sj.bjc.6690505

**Published:** 1999-04

**Authors:** M Sartor, H Steingrimsdottir, F Elamin, J Gäken, S Warnakulasuriya, M Partridge, N Thakker, N W Johnson, M Tavassoli

**Affiliations:** Oral Oncology Group, Departments of Oral Medicine and Pathology, andMolecular Medicine, Molecular Oncology Group, Department of Oral and Maxillofacial Surgery, The Rayne Institute,King's College School of Medicine and Dentistry, 125 Coldharbour Lane, London SE5 9NU, UK; Department of Medical Genetics, University of Manchester, St Mary's Hospital, Manchester M13 0JH, UK

**Keywords:** oral cancer, p16/MTS1, cyclin D1, retinoblastoma, G1 checkpoint

## Abstract

One of the most important components of G1 checkpoint is the retinoblastoma protein (pRB^110^). The activity of pRB is regulated by its phosphorylation, which is mediated by genes such as *cyclin D1* and *p16*/*MTS1*. All three genes have been shown to be commonly altered in human malignancies. We have screened a panel of 26 oral squamous cell carcinomas (OSCC), nine premalignant and three normal oral tissue samples as well as eight established OSCC cell lines for mutations in the *p16*/*MTS1* gene. The expression of *p16*/*MTS1*, *cyclin D1* and pRB^110^ was also studied in the same panel. We have found *p16*/*MTS1* gene alterations in 5/26 (19%) primary tumours and 6/8 (75%) cell lines. Two primary tumours and five OSCC cell lines had p16/MTS1 point mutations and another three primary and one OSCC cell line contained partial gene deletions. Six of seven p16/MTS1 point mutations resulted in termination codons and the remaining mutation caused a frameshift. Western blot analysis showed absence of p16/MTS1 expression in 18/26 (69%) OSCC, 7/9 (78%) premalignant lesions and 8/8 cell lines. One cell line, H314, contained a frameshift mutation possibly resulting in a truncated p16/MTS1 protein. pRB was detected in 14/25 (56%) of OSCC but only 11/14 (78%) of these contained all or some hypophosphorylated (active) pRB. In premalignant samples, 6/8 (75%) displayed pRB, and all three normal samples and eight cell lines analysed contained RB protein. p16/MTS1 protein was undetectable in 10/11 (91%) OSCCs with positive pRB. Overexpression of cyclin D1 was observed in 9/22 (41%) OSCC, 3/9 (33%) premalignant and 8/8 (100%) of OSCC cell lines. Our data suggest p16/MTS1 mutations and loss of expression to be very common in oral cancer cell lines and less frequent in primary OSCC tumours. A different pattern of p16/MTS1 mutations was observed in OSCC compared to other cancers with all the detected p16/MTS1 mutations resulting in premature termination codons or a frameshift. The RB protein was expressed in about half (44%) of OSCCs and its expression inversely correlated with p16/MTS1 expression. In conclusion, we show that abnormalities of the RB pathway are a common mechanism of oral carcinogenesis. © 1999 Cancer Research Campaign


*British Journal of Cancer* (1999) **80**(1/2), 79–86
© 1999 Cancer Research Campaign
Article no. bjoc.1998.0325

### Role of p16/MTS1, cyclin D1 and RB in primary oral cancer and oral cancer cell lines

**M Sartor[1], H Steingrimsdottir[1], F Elamin[1], J Gäken[2], S Warnakulasuriya[1], M Partridge[3], N Thakker[4], NW Johnson[1] and M Tavassoli[1]**

Oral Oncology Group, Departments of [1]Oral Medicine and Pathology and [2]Molecular Medicine, [3]Molecular Oncology Group, Department of Oral and Maxillofacial Surgery, The Rayne Institute, King's College School of Medicine and Dentistry, 125 Coldharbour Lane, London SE5 9NU, UK; [4]Department of Medical Genetics, University of Manchester, St Mary's Hospital, Manchester M13 0JH, UK

**Summary** One of the most important components of G1 checkpoint is the retinoblastoma protein (pRB$^{110}$). The activity of pRB is regulated by its phosphorylation, which is mediated by genes such as *cyclin D1* and *p16*/*MTS1*. All three genes have been shown to be commonly altered in human malignancies. We have screened a panel of 26 oral squamous cell carcinomas (OSCC), nine premalignant and three normal oral tissue samples as well as eight established OSCC cell lines for mutations in the *p16*/*MTS1* gene. The expression of *p16*/*MTS1*, *cyclin D1* and pRB$^{110}$ was also studied in the same panel. We have found *p16*/*MTS1* gene alterations in 5/26 (19%) primary tumours and 6/8 (75%) cell lines. Two primary tumours and five OSCC cell lines had p16/MTS1 point mutations and another three primary and one OSCC cell line contained partial gene deletions. Six of seven p16/MTS1 point mutations resulted in termination codons and the remaining mutation caused a frameshift. Western blot analysis showed absence of p16/MTS1 expression in 18/26 (69%) OSCC, 7/9 (78%) premalignant lesions and 8/8 cell lines. One cell line, H314, contained a frameshift mutation possibly resulting in a truncated p16/MTS1 protein. pRB was detected in 14/25 (56%) of OSCC but only 11/14 (78%) of these contained all or some hypophosphorylated (active) pRB. In premalignant samples, 6/8 (75%) displayed pRB, and all three normal samples and eight cell lines analysed contained RB protein. p16/MTS1 protein was undetectable in 10/11 (91%) OSCCs with positive pRB. Overexpression of cyclin D1 was observed in 9/22 (41%) OSCC, 3/9 (33%) premalignant and 8/8 (100%) of OSCC cell lines. Our data suggest p16/MTS1 mutations and loss of expression to be very common in oral cancer cell lines and less frequent in primary OSCC tumours. A different pattern of p16/MTS1 mutations was observed in OSCC compared to other cancers with all the detected p16/MTS1 mutations resulting in premature termination codons or a frameshift. The RB protein was expressed in about half (44%) of OSCCs and its expression inversely correlated with p16/MTS1 expression. In conclusion, we show that abnormalities of the RB pathway are a common mechanism of oral carcinogenesis.

**Keywords:** oral cancer; p16/MTS1; cyclin D1; retinoblastoma; G1 checkpoint

Oral squamous cell carcinoma (OSCC) is the most common malignancy in South Asia. Carcinogens such as alcohol, tobacco and nitrosamines contained in areca nut are known to cause the development of most oral cancers but the molecular mechanisms involved in the malignant transformation of oral epithelial cells are still unclear (Johnson, 1991). Aberrations in the *p53* gene have been shown to be the most common genetic alterations in oral cancers (Wong et al, 1996). However, about 50% of oral cancers seem to have wild-type (wt) *p53*, suggesting that other genes may contribute to the development of oral malignancies. Recently, much attention has been focused on the role of the G1 checkpoint in human cancer. Apart from *p53*, the retinoblastoma (RB) gene product, pRB$^{110}$, is another key component of this checkpoint (Weinberg, 1995). pRB$^{110}$ was the first of the 'pocket' proteins to be characterized (Goodrich and Lee, 1993). The other known members of this family of proteins are p130 and p107 (Ewen et al, 1993; Mayol et al, 1993). pRB binds a number of viral proteins such as E7 from certain types of papillomaviruses (Li et al, 1993), large T antigen of SV40, E1A of certain adenoviruses (Wang et al, 1991) and IE2 of cytomegaloviruses (CMV) (Hagemeier et al, 1994). It also binds several cellular proteins including cyclin-dependent kinases and the E2F transcription factor (Bagchi et al, 1991; Chellappan et al, 1991), suggesting a very important role for RB in controlling cellular growth. pRB$^{110}$ activity is regulated by phosphorylation, which occurs in a cell cycle-dependent manner (Chen et al, 1989). Dephosphorylation of pRB$^{110}$ renders it active, leading to G1 arrest. The phosphorylation and inactivation of pRB is thought to be induced by successive waves of cyclins D1, D2 and D3 together with CDK4 or CDK6 and cyclin E together with CDK2. The function of these complexes is counteracted by the activity of cyclin-dependent kinase inhibitors (CDKIs) (reviewed by Hunter and Pines, 1994; Sherr, 1994). Two CDKI families are known, the prototype genes of these families being p16/CDKN2/MTS1 (Serrano et al, 1993) and p21/WAF1/CIP1 respectively. p21/WAF1 is thought to be a 'universal' CDKI, inhibiting the activity of both Cyclin D-CDK4/6 and Cyclin E-CDK2 complexes (Xiong et al, 1993) while p16/MTS1 only inhibits cyclin D1-CDK4/6 complexes (Serrano et al, 1993). The expression of p16/MTS1 is constantly low during most of the cell cycle, peaking with a slight increase at G1 phase (Tam et al, 1994;

*Received 17 July 1998*
*Revised 7 October 1998*
*Accepted 21 October 1998*

*Correspondence to:* M Tavassoli

Stone et al, 1995). Ectopic overexpression of p16/MTS1 has been shown to result in G1 arrest (Koh et al, 1995; Lukas et al, 1995; Serrano et al, 1996). This arrest is dependent on the presence of (wt) pRB (Medema et al, 1995).

p16/MTS1 is located on 9p21 in humans, a region commonly deleted in many tumour types (Fountain et al, 1992; Kamb et al 1994). As well as gene deletion (van der Riet et al, 1994; Reed et al., 1996), point mutations (Zhou et al, 1994; Liu et al, 1995; Arap et al, 1997), methylation (Gonzalez-Zulueta et al, 1995; Otterson et al, 1995; Shapiro et al, 1995) and the TAX protein of HTLV1 virus (Suzuki et al, 1996) have been found to inactivate p16/MTS1 in several tumour types including head and neck (Reed et al, 1996; Olshan et al, 1997; Papadimitrakopoulou et al, 1997).

Cyclin D1/PRAD1/BCL1 is located on 11q13 in humans, a region commonly amplified in several types of cancer (Berenson et al, 1989; Bartkova et al, 1995*a*, 1995*b*). As well as amplification, mutations which result in the stabilization of cyclin D1 protein have also been suggested to be a mechanism for the abnormal accumulation of cyclin D1 (Welcker et al, 1996). Ectopic expression of cyclin D1 results in the acceleration of the G1/S phase transition, showing that cyclin D1 is rate-limiting in this step (Jiang et al, 1993; Ohtsubo and Roberts 1993). The acceleration through the G1 checkpoint due to cyclin D1 overexpression has been shown to result in an increase in genomic instability (Zhou et al, 1996).

Despite the importance of $pRB^{110}$ in regulating the G1 checkpoint, mutations in $pRB^{110}$ are uncommon in head and neck squamous cell carcinomas (Yoo et al, 1994). This suggests that not only $pRB^{110}$, but also the proteins regulating RB function, may be involved in carcinogenesis of head and neck epithelium.

In this study we have assessed aberrations in genes upstream of RB including p16/MTS1, cyclin D1 as well as $pRB^{110}$ itself in a panel of OSCC and premalignant oral lesions as well as OSCC cell lines. Our data suggest that loss of expression of p16/MTS1 is very common in OSCC. Also, cyclin D1 was frequently found to be overexpressed in malignancies of the oral cavity. While the RB gene itself seems to be less prone to alterations in this type of cancer its expression and/or activity is altered by other proteins such as p16/MTS1, cyclin D1 and possibly E7 of HPV-16.

### MATERIALS AND METHODS

### Sample selection

Fresh oral biopsies were collected from 38 patients with lesions clinically and histologically diagnosed as OSCCs or premalignant mucosal lesions from six hospitals in South East England. Three pathologically normal specimens were also included. The total panel consisted of 22 male and 16 female and their mean age was 65.6 ± 2.1 years. Histology confirmed that 26 of these were malignant OSCC; nine were keratoses with varying grades of dysplasia (Tables 1 and 2). OSCC were histologically graded as well, moderate and poorly differentiated and the severity of dysplasia as mild, moderate or severe by the Smith and Pindborg criteria (1969). The samples were stored in the gas liquid phase of liquid nitrogen until further use. The oral cancer cell lines were gifts from Profs Stephen Prime, Bristol (H103, H157, H314, H357, H376, H400) and Barry Gusterson, Sutton (HN5 and HN6). The cells were grown under the conditions which have been previously described (Bartkova et al, 1995*a*; Yeudall et al, 1995).

### DNA preparation and mutational analysis

The p16/MTS1 polymerase chain reaction (PCR) was performed using primers; exon 1 (sense: 5′-CGG CTG CGG AGA GGG GGA GA-3′, antisense: 5′-CCG CTG CAG ACC CTC TAC CCA CCT-3′), exon 2 part 1 (sense: 5′-ACA AGC TTC CTT TCC GTC ATG CCG- 3′, antisense: 5′-CCA GGC ATC GCG CAC GTC CA-3′) exon 2 part 2 (sense: 5′-TTC CTG GAC ACG CTG GTG GT-3′, antisense: 5′-TCT GAG CTT TGG AAG CTC TCA G-3′) exon 3 (sense: 5′-CGC CTG TTT TCT TTC TGC CCT CTG-3′, antisense: 5′-GAA AGC GGG GTG GGT TGT GG-3′). Amplification of glyceraldehyde 3-phosphate dehydrogenase (GAPDH) was used as an internal PCR control. Oligonucleotide primers for GAPDH were (sense: 5′-AGT ACG CTG CAG GGC CTC ACT CC TT-3′, antisense 5′-AAG AGC CAG TCT CTG GCC CCA GCC A-3′). The PCR reaction consisted of 1 µl of DNA extract, 25 pmol of each primer, 1X PCR Promega Formula buffer containing 1.5 mM magnesium, 200 µM dNTPs, 5% dimethyl sulphoxide (DMSO) and 1.5 U *Taq* polymerase (Advanced Biotechnology) in a total volume of 50 µl. The PCR mix was denatured at 94°C for 5 min, followed by 30 cycles (94°C, 30 s; 62°C, 1 min and 72°C, 1 min).

DNA was isolated from 2 × 6 µm cryostat sections from frozen specimens for which adjacent sections were examined by microscopy for assessment of the presence of adequate tumour tissue and the proportion of stromal tissue. All OSCC samples used for DNA extraction showed > 60% tumour tissue in each case. Cellular DNA was extracted as we have previously described (Steingrimsdottir et al, 1997). Briefly, the samples were lysed in sodium dodecyl sulphate (SDS) buffer [100 mM sodium chloride (NaCl), 10 nM Tris-HCl, 25 mM EDTA, 0.5% SDS pH 8.0] and were incubated with 0.1 mg $ml^{-1}$ proteinase K for approximately 5 h. The lysate was then treated with an equal volume of phenol–chloroform–isoamyl alcohol pH 8.0 (25:24:1). DNA was recovered from the aqueous phase by the addition of 2 volumes of ethanol and was stored in 100 µl TE containing 2 µg RNAse A. For each PCR reaction, 1 µl DNA extract equal to approximately 100 ng was used.

### PCR amplification and SSCP analysis of the *p16/MTS1* gene

For SSCP analysis, the PCR products were labelled after 15 cycles by the addition of 1 µCi [$\alpha$-$^{32}$P]dCTP. A total of 3 µl of the labelled PCR products were diluted with 4 µl of 95% deionized formamide containing bromophenol blue–xylene cyanol, 3.7 µl EDTA (0.5 M) and 26.25 µl water. The PCR mixtures were denatured for 5 min at 95°C, and were snap frozen by placing in liquid nitrogen. The denatured products (5 µl) were loaded on an 8% non-denaturing polyacrylamide gel electrophoresis (PAGE) gel and separated using 1 × TBE in the upper chamber (cathode) and 2 × TBE in the lower chamber (anode). Electrophoresis was carried out at 350 volts at room temperature overnight. The gels were dried and exposed to X-ray film developing after 16 h.

For sequencing, 40 µl of the PCR products were electrophoresed in a 1% low melting point agarose gel and the target bands were excised from the gel. The selected agarose fragments were sliced and digested with Agarase (B-Agarase I, Calbiochem) according to the manufacturer’s recommendation. The purified DNA products were then sequenced directly using both 5′ and 3′ primers together with dye terminators in an ABI 373A automated sequencer.

 © Cancer Research Campaign 1999

### Expression analysis

For Western blotting, samples were lysed in Laemmli sample buffer (Laemmli, 1970), which were then boiled and resolved by SDS-PAGE, 6–12.5%. The gel was transferred to 0.45 μM nitrocellulose membrane (Schleicher and Schuell) as previously described (Towbin et al, 1992). Probing of the blot and detection of the antibodies were performed according to the manufacturer's instructions for the enhanced chemiluminescent reaction (ECL, from Amersham). The membrane was blocked for 1 h in 4% milk powder containing 1% bovine serum albumin (BSA) in TBST buffer (20 mM Tris pH 8.0, 150 mM NaCl, 0.05% Tween-20). For p16, the antibody used was DCS-50 and for cyclin D1, DCS-6 (both gifts of Dr Jiri Bartek and Dr Gordon Peters). Both p16 and cyclin D1 antibodies were diluted 1:1000 in TBST. For pRB, the antibody used was 14001A (Pharmingen), diluted 1:500 in TBST. Secondary antibodies tagged to horseradish peroxidase were detected using the enhanced chemiluminescence method according to the manufacturer's instructions (ECL, from Amersham). p16-positive cell line MDAMB 468 and cyclin D1-positive cell line MCF7 were used as positive controls.

### RESULTS

### *p16/MTS1* gene deletion/mutation analysis

DNA from 26 OSCC tumours, nine premalignant and three normal samples as well as eight OSCC cell lines was analysed by PCR. Exons 1α, 2 and 3 of p16/MTS1 were independently amplified using GAPDH primers in the same reactions as an internal control. Because of the large size of p16/MTS1 exon 2 (305 bp), this was amplified as two separate fragments (parts I and II).

Samples 13, 14 and 24 failed to amplify the 218 bp fragment expected for exon 1α. These samples amplified a 474 bp fragment corresponding to the GAPDH fragment, albeit a weaker band was observed for 24 (Figure 1). These results suggested that at least part of exon 1α is deleted in these three tumours (Figure 1).

To examine the *p16/MTS1* gene mutations the PCR products were then analysed by single-stranded conformation polymorphism (SSCP) and samples that showed abnormal shifts on the SSCP gel were subsequently sequenced directly. Point mutations were detected in 2/26 (8%) of the primary oral tumours, 6 and 15. Both of these were non-sense mutations at codon 58 (CGA>TGA, arg>term). Sample 6 also showed a transition at codon 148 (GCG>ACG, ala>thr), which is a known p16/MTS1 polymorphism (Tables 1 and 2).

Analysis of the *p16/MTS1* gene in the cell lines by PCR amplification showed deletion of exon 3 in one cell line, HN6. Point mutations in the *p16/MTS1* gene were found in 5/8 (63%) of OSCC cell lines (Tables 1 and 2). We found non-sense mutations at codons 58 (CGA>TGA, arg>term) in H103 and H357, and codon 80 (CGA>TGA, arg>term) in H157. In HN5, codon 88 was mutated substituting GAG>TAG, glu>term. In H314, the deletion of a single G at codon 69 caused a frameshift. This frameshift results in several termination codons in the reading frame, the first one being in codon 119. None of the premalignant or normal samples showed either deletions or point mutations in the *p16/MTS1* gene.

### p16/MTS1 expression

Expression of p16/MTS1 was analysed in primary samples and OSCC cell lines by Western blotting, using the DCS-50 antibody. The expression results are summarized in Tables 1 and 2. Absence of p16 was observed in 18/26 (69%) OSCC, whilst 8/26 (31%) OSCCs and 2/9 (22%) premalignant lesions expressed p16/MTS1 (Table 1 and Figure 2). All 8/8 (100%) cell lines lacked the expression of p16/MTS1. Sample 6, which had a p16/MTS1 mutation resulting in a termination codon (Tables 1 and 2), did not express normal size p16/MTS1. However, sample 15, which had the same termination mutation as 6, expressed p16/MTS1 protein. Also, sample 24 with exon 1 deletion lacked p16/MTS1 expression, while both samples 13 and 14 with a similar deletion expressed moderate to high levels of p16/MTS1. Western blot analysis detected a protein of approximately 25 kDa in the H314 cell line using C-20 p16/MTS1 antibody (Santa Cruz). However, such protein was not detected using another p16/MTS1 antibody, DCS-50.

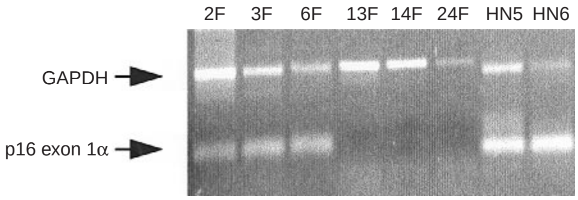

**Figure 1** Analysis of p16/MTS1 exon 1α by PCR. Exon 1α of p16/MTS1 gene was co-amplified with the internal control GAPDH and 10 μl of PCR products were separated on a 2% agarose gel. The bands corresponding to the predicted molecular weights of the GAPDH (474 bp) and p16/MTS1 exon 1α (218 bp) amplicons are indicated on the gel by arrows. Lane numbers correspond to the sample references in Table 1

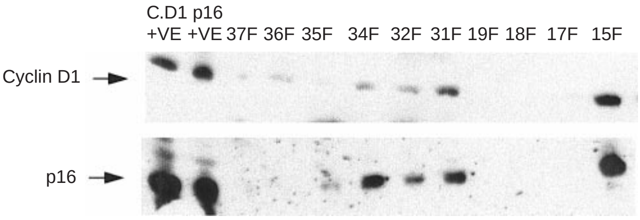

**Figure 2** Western blot analysis of cyclin D1 and p16 in primary oral lesions and oral cancer cell lines. Total cell or tissue lysates were separated in a 12.5% SDS-PAGE, transferred to a nitrocellulose membrane and the membrane was hybridized to antibodies DCS-50 for p16 and DCS-6 for cyclin D1. The lanes indicated +C. D1 for MCF-7 cell lysate as cyclin D1-positive control; +p16 for MDAMB 453 cell lysate as p16-positive control. The rest of the lanes correspond to the sample references in Table 1

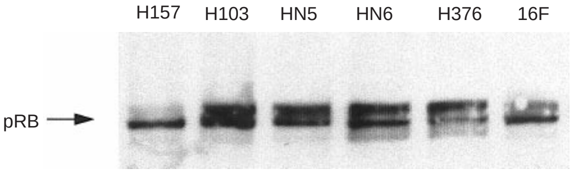

**Figure 3** Western blot analysis of pRB in oral samples. Total cell or tissue lysates were separated on a 6% SDS-PAGE and the membrane was hybridized to antibody 14001A. Samples are marked according to the sample references in Table 1

© Cancer Research Campaign 1999

**Table 1** Comparison of p16/MTS1 mutation analysis and expression of p16, cyclin D1 and pRB in primary oral lesions

| Sample number | Sample histology | p16 Mutation analysis | p16 expression | Cyclin D1 expression | pRB expression | pRB phosphorylation status |
|---|---|---|---|---|---|---|
| 2 | OSCC | WT | – | NA | + | RB$^{105}$ |
| 3 | OSCC | WT | – | – | + | RB$^{105}$ |
| 4 | OSCC | WT | – | NA | + | RB$^{105}$ |
| 6 | OSCC | αα58;CGA>TGA<br>αα148;GCG>ACG | – | NA | – | no RB |
| 8 | OSCC | WT | + | + | + | RB$^{110}$ |
| 9 | OSCC | WT | – | + | – | no RB |
| 10 | OSCC | WT | ++ | + | + | RB$^{110}$ |
| 11 | OSCC | WT | + | – | + | RB$^{105/110}$ |
| 13 | OSCC | deletion exon 1 | + | – | – | no pRB |
| 14 | OSCC | deletion exon 1 | ++ | + | – | no RB |
| 15 | OSCC | αα58;CGA>TGA | ++ | + | NA | NA |
| 17 | OSCC | WT | – | – | + | RB$^{105}$ |
| 18 | OSCC | WT | – | – | + | RB$^{105}$ |
| 19 | OSCC | WT | – | – | – | no RB |
| 20 | OSCC | WT | – | – | + | RB$^{105}$ |
| 22 | OSCC | WT | – | – | + | RB$^{105/110}$ |
| 23 | OSCC | WT | – | | – | no pRB |
| 24 | OSCC | deletion exon 1 | – | – | + | RB$^{110}$ |
| 26 | OSCC | WT | – | + | + | RB$^{105}$ |
| 27 | OSCC | WT | – | + | + | RB$^{105}$ |
| 30 | OSCC | WT | – | – | – | no RB |
| 31 | OSCC | WT | + | + | – | no RB |
| 32 | OSCC | WT | + | – | – | no RB |
| 36 | OSCC | WT | – | – | – | no RB |
| 37 | OSCC | WT | – | – | – | no RB |
| 42 | OSCC | WT | – | + | + | RB$^{105/110}$ |
| 1 | pre-cancer | WT | – | – | + | RB$^{105/110}$ |
| 12 | pre-cancer | WT | + | + | + | RB$^{105}$ |
| 16 | pre-cancer | WT | – | – | + | RB$^{105/110}$ |
| 25 | pre-cancer | WT | – | + | NA | NA |
| 34 | pre-cancer | WT | + | + | – | no RB |
| 35 | pre-cancer | WT | – | – | + | RB$^{105}$ |
| 38 | pre-cancer | WT | – | – | + | RB$^{105}$ |
| 40 | pre-cancer | WT | – | – | + | RB$^{105}$ |
| 43 | pre-cancer | WT | – | – | – | no RB |
| 33 | normal | WT | + | – | + | RB$^{105}$ |
| 39 | normal | WT | – | | + | RB$^{105}$ |
| 41 | normal | WT | – | – | + | RB$^{105}$ |

The pRB expression column refers to '+' for samples which expressed either, one or both hypo- and hyperphosphorylated forms of pRB. The pRB phosphorylation column describes the phosphorylation status of pRB. Samples expressing only dephosphorylated pRB are denoted as 'RB$^{105}$', only phosphorylated pRB as 'RB$^{110}$', no expression as 'no pRB' and expression of both forms as 'RB$^{105/110}$'. WT, wild-type sequence, NA, not analysed.

### Cyclin D1 and RB expression

Western blot analysis using DCS-6 antibody detected high levels of cyclin D1 in 9/22 (41%) of the tumours, 3/9 (33%) potential malignancies and in 100% (8/8) of the OSCC cell lines. Both normal samples analysed expressed low levels of cyclin D1.

pRB was expressed in 14/25 (56%) of the cancers, 6/8 (75%) premalignant lesions and all three normals. The level of pRB phosphorylation varied in different samples. pRB was hyperphosphorylated in 3/14 malignant samples (8, 10 and 24), indicating the presence of an inactive pRB. Surprisingly, all (8/8) cell lines expressed pRB but in one cell line, H157, mainly the unphosphorylated form of pRB was detected (Tables 1 and 2 and Figures 2 and 3).

### Comparison between pRB, cyclin D1 and p16/MTS1 expression

The pRB expression results obtained by Western blotting were compared to those for p16/MTS1 and cyclin D1. We found an inverse association between the expression of p16/MTS1 and the presence of pRB. Lack of p16/MTS1 expression was observed in 10/11 (91%) OSCCs which contained active pRB. Also 5/6 (83%) of the premalignant lesions which were pRB-positive lacked p16/MTS1 protein (Table 3) and (8/8) 100% cell lines expressed pRB but no p16/MTS1. Comparison between cyclin D1 and p16/MTS1 showed 10/13 (77%) OSCCs and 6/6 premalignant samples did not express either Cyclin D1 or p16/MTS1 (Table 4). Cyclin D1 and pRB expression also showed some correlation: 5/6

*© Cancer Research Campaign 1999*

**Table 2** Comparison of p16/MTS1 mutation/deletion and expression of p16, cyclin D1 and pRB in OSCC cell lines

| Cell line | p16 mutation status | p16 expression | Cyclin D1 expression | pRB expression | pRB phosphorylation status |
|---|---|---|---|---|---|
| HN5 | αα88; GAG>TAG | – | ++ | + | $RB^{105/110}$ |
| HN6 | deletion exon 3 | – | ++ | + | $RB^{105/110}$ |
| H103 | αα58; CGA>TGA | – | ++ | + | $RB^{105/110}$ |
| H157 | αα80; | – | ++ | + | $RB^{105}$ |
| H314 | αα69; deletion of G | – | ++ | + | $RB^{105/110}$ |
| H357 | αα58; CGA>TGA | – | ++ | + | $RB^{105/110}$ |
| H376 | WT | – | ++ | + | $RB^{105/110}$ |
| H400 | WT | – | ++ | + | $RB^{105/110}$ |

The pRB expression column refers to '+' for samples which expressed either, one or both hypo- and hyperphosphorylated forms of pRB. The pRB phosphorylation column describes the phosphorylation status of pRB. Samples expressing only dephosphorylated pRB are denoted as '$RB^{105}$', only phosphorylated pRB as '$RB^{110}$', no expression as 'no pRB' and expression of both forms as '$RB^{105/110}$'. WT, wild-type sequence, NA, not analysed.

**Table 3** Comparison between expression of p16/MTS1 and active pRB

| Samples | Positive pRB | | Negative pRB | |
|---|---|---|---|---|
| | Positive p16 | Negative p16 | Positive p16 | Negative p16 |
| OSCC | 1/11 (9%) | 10/11 (91%) | 6/14 (43%) | 8/14 (57%) |
| Pre-cancer | 1/6 (17%) | 5/6 (83%) | 1/2 (50%) | 1/2 (50%) |

pRB was scored as active if either only the hypophosphorylated or both hypo- and hyperphosphorylated forms were detected.

**Table 4** Comparison between expression of cyclin D1 p16/MTS1 by Western blotting

| Samples | Positive cyclin D1 | | Negative cyclin D1 | |
|---|---|---|---|---|
| | Positive p16 | Negative p16 | Positive p16 | Negative p16 |
| OSCC | 4/9 (56%) | 4/9 (44%) | 3/13 (23%) | 10/13 (77%) |
| Pre-cancer | 2/3 (67%) | 1/3 (33%) | 0/6 (0%) | 6/6 (100%) |

(83%) of the premalignant lesions expressed pRB but no cyclin D1 while only 6/9 (67%) of the OSCCs showed that pattern of expression (Table 5). The number of normal samples in this study was too small to make any correlation.

### DISCUSSION

In order to understand the role of the RB pathway in oral cancer we studied the expression of pRB as well as its upstream regulators p16/MTS1 and cyclin D1 in primary oral lesions and oral cancer cell lines. We also analysed p16/MTS1 mutations in the same panel. To our knowledge, few studies have analysed the expression of these three key proteins in primary OSCCs (Andl et al, 1998).

In primary OSCCs, p16/MTS1 point mutations/deletions occurred relatively frequently (19%) but in the cell lines the *p16/MTS1* gene was altered with a much higher frequency (75%). No *p16/MTS1* gene alterations were found in premalignant or normal samples. This large difference in the frequencies, also reported by others (Zhang et al, 1994), could be due to instability of the *p16/MTS1* gene in the cell lines maintained for long duration in culture. Mutations in the *p16/MTS1* gene could also confer a growth advantage to cells and therefore cells with such mutations are selected for establishing lines. Another reason for the lower incidence of p16/MTS1 mutations in primary tumours could be due to the heterogeneity of the tissues analysed. The presence of normal cells in the specimens can mask the detection of p16/MTS1 point mutations/deletions.

Mechanisms other than gene mutation have also been shown to be responsible for down-regulation of the *p16/MTS1* gene (Hara et al, 1996). DNA methylation has been shown to play an important role in silencing *p16/MTS1* gene transcription (Otterson et al, 1995). In this study, 16/26 (62%) OSCCs, 5/9 (56%) premalignant

**Table 5** Comparison between expression of cyclin D1 and active pRB

| Samples | Positive pRB | | Negative pRB | |
|---|---|---|---|---|
| | Positive cyclin D1 | Negative cyclin D1 | Positive cyclin D1 | Negative cyclin D1 |
| OSCC | 3/9 (33%) | 6/9 (67%) | 5/12 (42%) | 7/12 (58%) |
| Pre-cancer | 1/6 (17%) | 5/6 (83%) | 1/2 (50%) | 1/2 (50%) |

pRB was scored as active if either only the hypophosphorylated or both hypo- and hyperphosphorylated forms were observed.

© Cancer Research Campaign 1999 

lesions and 2/8 cell lines which had no detectable p16/MTS1 mutation, lacked p16/MTS1 protein. This indicates that epigenetic mechanisms such as DNA methylation could be responsible for inducing gene silencing in these samples.

In three primary tumours p16/MTS1 exon 1α was not amplified despite amplification of exons 2 and 3 as well as the GAPDH gene. We interpreted this to be a partial deletion of *p16/MTS1* gene. Surprisingly, two of these three samples expressed p16/MTS1 protein (Tables 1 and 2). This could be due to the presence of small deletions or point mutations preventing primer annealing and hence gene amplification, but not affecting the reading frame and expression of *p16/MTS1* gene. Alternatively, this discrepancy could be due to the use of different sections taken from different parts of the tumour for use in the PCR and Western blot analyses.

In sample 24, which failed to amplify exon 1α, the level of GAPDH was also lower, which was probably due to the presence of less DNA in this particular sample available for the PCR reaction. We checked and confirmed the partial deletions in the *p16/MTS1* gene in these three tumour samples in three independent PCR reactions. However, we are aware of the limitations of the semiquantitative PCR technique for deletion detection, therefore we are cautious interpreting our PCR results. To minimize artefacts, only sections with more than 60% malignant cells were used for DNA and protein analysis. The option of tissue microdissection was also considered, but preliminary experiments showed that this process greatly increases the risk of tissue contamination caused by the extensive manipulations performed on the tissues. The frequency of deletions within or encompassing the *p16/MTS1* gene has been found to vary greatly. For example, one study (Reed et al, 1996) showed homozygous deletions of 9p in 67% of head and neck tumour samples, whilst Zhang et al (1994) could not detect any p16/MTS1 deletions in 68 head and neck tumours that they analysed. Also, it has been shown that deletions on 9p do not necessarily correlate with loss of p16/MTS1 expression. Indeed, it has been shown that loss of p16/MTS1 expression can occur at a much higher frequency than deletion of the *p16/MTS1* gene (Gonzalez-Zulueta et al, 1995). The converse is also true: deletions in 9p do not necessarily affect p16/MTS1 expression (Cheng et al, 1994; Farrell et al, 1997).

Deletions encompassing exon 1α could affect other genes upstream of *p16/MTS1*, such as the CDKI p15/MTS2 (Hannon and Beach, 1994) or p19$^{ARF}$ (Quelle et al, 1995, 1997). Two recent papers discuss the importance of p19$^{ARF}$ in the regulation of the p53 pathway (Pomerantz et al, 1998; Zhang et al, 1998). p19$^{ARF}$ can suppress cellular proliferation in cells bearing wt p53, but not otherwise (Kamijo et al, 1997). This is thought to occur by virtue of p19$^{ARF}$ ability to destabilize the MDM2 protein (Pomerantz et al, 1998; Zhang et al, 1998). The MDM2 protein has also been shown to play a role in the pRB regulated cell cycle control (Martin et al, 1995; Xiao et al, 1995). Alterations in p19$^{ARF}$ protein as a result of p16/MTS1 mutations could affect its function, leading to pRB deregulation and development of cancer.

Interestingly, we found that all but one of the *p16/MTS1* gene point mutations resulted in a termination codon. The remaining mutation identified in the cell line H314 created a frameshift. This frameshift results in several termination codons after codon 119, thus presumably a truncated p16/MTS1 protein is produced. This observation shows that the spectrum of p16/MTS1 mutation in oral cancer is different to other tumour types and may be related to carcinogens contained in the aetiological factors for oral cancer such as tobacco and alcohol. For example, most p16/MTS1 mutations in glioblastomas (Kyritsis et al, 1996), oesophageal (Mori et al, 1994), lung, leiomyosarcoma, chondrosarcoma, prostate and non-small cell lung cancer cell lines analysed are mis-sense mutations (Liu et al, 1995; Ruas and Peters, 1998). However, melanomas have also been shown to frequently contain non-sense mutations in the *p16/MTS1* gene (Liu et al, 1995). Of the OSCCs analysed by Zhang et al (1994) only a small percentage contained non-sense mutations in the *p16/MTS1* gene, though in other studies no non-sense mutations were found in the OSCCs analysed (Yoshida et al, 1995).

Western blot analysis detected pRB in 14/25 (56%) OSCCs, 6/8 (75%) premalignant samples as well as all 8 cell lines. Down-regulation of pRB expression occurred in about half of the OSCC primary tumours.

The function of pRB has shown to be normally regulated by phosphorylation (Chen et al, 1989). Hyperphosphorylation of pRB, which is controlled by cyclin D1-CDK4/6 renders pRB inactive. Cyclin-dependent kinase inhibitors such as p16/MTS1 are responsible for inhibiting pRB phosphorylation and thus induce pRB activity. To understand the role of pRB in the control of cell growth, it is important to examine not only the level but also the phosphorylation status of RB protein. We examined the status of RB phosphorylation in the panel of OSCCs studied and observed that in most samples both hyper- and hypophosphorlated forms of pRB were present. The samples which expressed both hypo- and hyper- or only hypophosphorylated pRB were classed as samples containing active pRB; the samples containing only hyperphosphorylated pRB were classed as having inactive pRB.

To understand the association between different components of the pRB pathway, we compared the status of p16, pRB and cyclin D1. In normal cells both p16 and cyclin D1 are expressed at low levels throughout the cell cycle. p16 is known to slightly peak at entry into S phase. Thus in non-malignant cells low or undetectable levels of cyclin D1 and p16 is expected. The absence or high steady state levels observed in some tumours therefore indicates the presence of abnormal regulatory mechanisms.

The results shown above suggested an inverse relationship (91%) between the presence of pRB and p16/MTS1 proteins (Table 2). Such correlation has previously been reported by Parry et al (1995) who have shown absence of pRB function to result in the accumulation of p16. However, our data suggest that the lack of p16/MTS1 expression is most likely due to its gene aberrations rather than due to inadequate regulation by pRB.

Cyclin D1 overexpression was found in 9/21 (43%) OSCC and in 3/9 (33%) premalignant samples. HN5 and HN6 have been previously reported not to bear amplification of the *cyclin D1* gene (Bartkova et al, 1995*b*). However, when compared to the positive control cell line MCF7, comparable expression of cyclin D1 was detected in HN5 and HN6. Thus, the expression of cyclin D1 in these cell lines was recorded as high. Surprisingly, 100% of the OSCC cell lines showed high levels of cyclin D1 expression. These results suggest that cyclin D1 overexpression is very common and possibly an early event in oral carcinogenesis. Other studies have shown moderate overexpression of cyclin D1 in up to 40% of OSCC cell lines (Timmermann et al, 1997). Also, amplification of the *cyclin D1* gene has been observed in 25% of oral dysplasias (Kyomoto et al, 1997). Overexpression of cyclin D1 in

 © Cancer Research Campaign 1999

our samples could be due to gene amplification. Although we have not examined cyclin D1 amplification, high frequency of *cyclin D1* gene amplification (between 30 and 50%) in head and neck cancers has been previously reported (Bartkova et al, 1995*b*).

When cyclin D1 expression was compared to pRB we found that 6/9 (67%) malignant tumours and 5/6 (83%) premalignant lesions had pRB expression but lacked cyclin D1. Whether such correlation bears any significance is not known.

The cyclin-dependent kinases 4 and 6 are dependent on cyclin D1 for their activity and regulate the activity of pRB by phosphorylation. Two recent reports have shown increased activity of CDK4 and/or 6 in OSCC cell lines compared to normal keratinocytes (Patel et al, 1997; Timmermann et al, 1997). It would have been desirable to include such an analysis in this study; however, these functional studies were not feasible with the small amounts of tissue available from our primary tumours.

In this study absence of cyclin D1 expression in 6/6 premalignant and 10/13 (77%) OSCC samples was generally accompanied by no p16/MTS1 expression. This suggests that both p16/MTS1 and cyclin D1 are tightly regulated in wild-type cells and that deregulation of one of the components of the G1 checkpoint leads to rapid accumulation of other abnormalities.

Infection with human papillomavirus can result in the down-regulation of pRB by virtue of its interaction with the E7 viral gene product (Li et al, 1993). Comparison of the presence of HPV 16 (Elamin et al, 1998) and pRB protein expression revealed that of the 15 samples which were HPV 16-positive, eight did not express pRB, two expressed only hyperphosphorylated pRB, four expressed only hypophosphorylated pRB and one OSCC sample expressed both hypo- and hyperphosphorylated pRB. The lack of functional pRB in 10/16 (63%) samples suggests that inactivation of pRB by E7 could be a major event in OSCC development.

In conclusion, we found deregulated expression of some of the components of the ‘pRB pathway’ to be consistent with the notion that this pathway is important for cell cycle control. Deregulation of pRB by aberrations in several cellular proteins such as p16/MTS1 and cyclin D1 and/or viral proteins such as E7 of HPV 16 can lead to the development of oral cancer.

### ACKNOWLEDGEMENTS

We are grateful to Profs Stephen Prime and Barry Gusterson for providing the cell lines, to Drs Jiri Bartek and Gordon Peters for the donation of DCS-50 and DCS-6 antibodies, to Dr Tim Crook for providing the p16/MTS1 primers as well as much helpful advice and to Mr John Penhallow for technical help. This work was supported by a grant from the Joint Research Committee, King’s Healthcare NHS Trust (Grant No. 15).

### REFERENCES

Andl T, Kahn T, Pfuhl A, Nicola T, Erber R, Conradt C, Klein W, Helbig M, Dietz A, Weidauer H, and Bosch FX (1998) Etiological involvement of oncogenic human papillomavirus in tonsillar squamous cell carcinomas lacking retinoblastoma cell cycle control. *Cancer Res* **58**: 5–13

Arap W, Knudsen ES, Wang JY, Cavenee WK and Huang HJ (1997) Point muatations can inactivate in vitro and in vivo activities of p16 (INK4a) /CDKN2A in human glioma. *Oncogene* **14**: 603–609

Bagchi S, Weinmann R and Raychaudhuri P (1991) The retinoblastoma protein copurifies with E2F-I, an E1A-regulated inhibitor of the transcription factor E2F. *Cell* **65**: 1063–1072

Bartkova J, Lukas J, Muller H, Strauss M, Gusterson B and Bartek J (1995*a*) Abnormal patterns of D-type cyclin expression and G1 regulation in human head and neck cancer. *Cancer Res* **55**: 949–956

Bartkova J, Lukas J, Strauss M and Bartek J (1995*b*) Cyclin D1 oncoprotein aberrantly accumulates in malignancies of diverse histogenesis. *Oncogene* **10**: 775–778

Berenson JR, Yang J and Mickel RA (1989) Frequent amplification of the bcl-1 locus in head and neck squamous cell carcinomas. *Oncogene* **4**: 1111–1116

Chellappan SP, Hiebert S, Mudryj M, Horowitz JM and Nevins JR (1991) The E2F transcription factor is a cellular target for the RB protein. *Cell* **55**: 1053–1061

Chen PL, Scully P, Shew JY, Wang JY and Lee WH (1989) Phosphorylation of the retinoblastoma gene product is modulated during the cell cycle and cellular differentiation. *Cell* **55**: 1193–1198

Cheng JQ, Jhanwar SC, Klein WM, Bell DW, Lee WC, Altomare DA, Nobori T, Olopade OI, Buckler AJ and Testa JR (1994) p16 alterations and deletion mapping of 9p21-p22 in malignant mesothelioma. *Cancer Res* **54**: 5547–5551

Elamin F, Steingrimsdottir H, Warnakulasuriya S, Johnson NW and Tavassoli M (1998) Prevalence of human papillomavirus infection in premalignant and malignant lesions of the oral cavity in U.K. subjects: A novel method of detection. *Eur J Cancer* **34**: 191–197

Ewen ME, Sluss HK, Sherr CJ, Matsushime H, Kato J and Livingston DM (1993) Functional interactions of the retinoblastoma protein with mammalian D-type cyclins. *Cell* **73**: 487–497

Farrell WE, Simpson DJ, Bicknell JE, Talbot AJ, Bates AS and Clayton RN (1997) Chromosome 9p deletions in invasive and noninvasive nonfunctional pituitary adenomas: the deleted region involves markers outside of the MTS1 and MTS2 genes. *Cancer Res* **57**: 2703–2709

Fountain JW, Karayiorgou M, Ernstoff MS, Kirkwood JM, Vlock DR, Titus Ernstoff L, Bouchard B, Vijayasaradhi S, Houghton AN, Lahti J, Kidd VJ, Housman DE and Dracopoli NC (1992) Homozygous deletions within human chromosome band 9p21 in melanoma. *Proc Natl Acad Sci USA* **89**: 10557–10561

Gonzalez-Zulueta M, Bender CM, Yang AS, Nguyen T, Beart RW, Van Tornout JM and Jones PA (1995) Methylation of the 5′ CpG island of the p16/CDKN2 tumor suppressor gene in normal and transformed human tissues correlates with gene silencing. *Cancer Res* **55**: 4531–4535

Goodrich DW and Lee WH (1993) Molecular characterization of the retinoblastoma susceptibility gene. *Biochim Biophys Acta* **1155**: 43–61

Hagemeier C, Caswell R, Hayhurst G, Sinclair J and Kouzarides T (1994) Functional interaction between the HCMV IE2 transactivator and the retinoblastoma protein. *EMBO J* **13**: 2897–2903

Hannon GJ, and Beach D (1994) p15INK4B is a potential effector of TGF-beta-induced cell cycle arrest [see comments]. *Nature* **371**: 257–261

Hara E, Smith R, Parry D, Tahara H, Stone S and Peters G (1996) Regulation of p16 (CDKN2) expression and its implications for cell immortalization and senescence. *Mol Cell Biol* **16**: 859–867

Hunter T and Pines J (1994) Cyclins and cancer. II: Cyclin D and CDK inhibitors come of age [see comments]. *Cell* **79**: 573–582

Jiang W, Kahn SM, Zhou P, Zhang YJ, Cacace AM, Infante AS, Doi S, Santella RM and Weinstein IB (1993) Overexpression of cyclin D1 in rat fibroblasts causes abnormalities in growth control, cell cycle progression and gene expression. *Oncogene* **8**: 3447–3457

Johnson NW (1991) A global view of the epidemiology of oral cancer. In *Oral Cancer*, Johnson NW (ed), pp. 3–26 Cambridge University Press: Cambridge

Kamb A, Gruis NA, Weaver Feldhaus J, Liu Q, Harshman K, Tavtigian SV, Stockert E, Day RS III, Johnson BE and Skolnick MH (1994) A cell cycle regulator potentially involved in genesis of many tumor types [see comments]. *Science* **264**: 436–440

Kamijo T, Zindy F, Roussel MF, Quelle DE, Downing JR, Ashmun RA, Grosveld G and Sherr CJ (1997) Tumour suppression at the mouse INK4a locus mediated by the alternative reading frame product $p19^{ARF}$. *Cell* **91**: 649–659

Koh J, Enders GH, Dynlacht BD and Harlow E (1995) Tumour-derived p16 alleles encoding proteins defective in cell-cycle inhibition. *Nature* **375**: 506–510

Kyomoto R, Kumazawa H, Toda Y, Sakaida N, Okamura A, Iwanaga M, Shintaku M, Yamashita T, Hiai H and Fukumoto M (1997) Cyclin-D1-gene amplification is a more potent prognostic factor than its protein over-expression in human head-and-neck squamous-cell carcinoma. *Int J Cancer* **74**: 576–581

Kyritsis AP, Zhang B, Zhang W, Xiao M, Takeshima H, Bondy ML, Cunningham JE, Levin VA and Bruner J (1996) Mutations of the p16 gene in gliomas. *Oncogene* **12**: 63–67

Laemmli UK (1970) Cleavage of structural proteins during the assembly of the head of bacteriophage T4. *Nature* **227**: 680–685

Li Y, Graham C, Lacy S, Duncan AM and Whyte P (1993) The adenovirus E1A-associated 130-kD protein is encoded by a member of the retinoblastoma gene family and physically interacts with cyclins A and E. *Genes Dev* **7**: 2366–2377

Liu Q, Neuhausen S, McClure M, Frye C, Weaver Feldhaus J, Gruis NA, Eddington K, Allalunis Turner MJ, Skolnick MH and Fujimura FK (1995) CDKN2 (MTS1) tumor suppressor gene mutations in human tumor cell lines. *Oncogene* **11**: 2455

Lukas J, Parry D, Agard L, Mann DJ, Bartkova J, Strauss M, Peters G, and Bartek J (1995) Retinoblastoma-protein-dependent cell-cycle inhibition by the tumour suppressor p16. *Nature* **375**: 503–506

Martin K, Trouche D, Hagemeier C, Sorensen TS, La Thangue NB and Kouzarides T (1995) Stimulation of E2F/DP1 transcriptional activity by MDM2 oncoprotein. *Nature* **375**: 691–694

Mayol X, Grana X, Baldi A, Sang N, Hu Q and Giordano A (1993) Cloning of a new member of the retinoblastoma gene family (pRb2) which binds to the E1A transforming domain. *Oncogene* **8**: 2561–2566

Medema RH, Herrera RE, Lam F and Weinberg RA (1995) Growth suppression by p16ink4 requires functional retinoblastoma protein. *Proc Natl Acad Sci USA* **92**: 6289–6293

Mori T, Miura K, Aoki T, Nishihira T, Mori S and Nakamura Y (1994) Frequent somatic mutation of the MTS1/CDK41 (multiple tumor suppressor / cyclin-dependent kinase 4 inhibitor) gene in esophageal squamous cell carcinoma. *Cancer Res* **54**: 3396–3397

Ohtsubo M and Roberts JM (1993) Cyclin-dependent regulation of G1 in mammalian fibroblasts. *Science* **259**: 1908–1912

Olshan AF, Weissler MC, Pei H, Conway K, Anderson S, Fried DB and Yarbrough WG (1997) Alterations of the p16 gene in head and neck cancer: frequency and association with p53, PRAD-1 and HPV. *Oncogene* **14**: 811–818

Otterson GA, Khleif SN, Chen W, Coxon AB and Kaye FJ (1995) CDKN2 gene silencing in lung cancer by DNA hypermethylation and kinetics of p16INK4 protein induction by 5-aza 2´ deoxycytidine. *Oncogene* **11**: 1211–1216

Papadimitrakopoulou V, Izzo J, Lippman SM, Lee JS, Fan YH, Clayman G, Ro JY, Hittelman WN, Lotan R, Hong WK and Mao L (1997) Frequent inactivation of p16INK4a in oral premalignant lesions. *Oncogene* **14**: 1799–1803

Parry D, Bates S, Mann DJ and Peters G (1995) Lack of cyclin D-Cdk complexes in Rb-negative cells correlates with high levels of p16INK4/MTS1 tumour suppressor gene product. *EMBO J* **14**: 503–511

Patel V, Jakus J, Harris CM, Ensley JF, Robbins KC and Yeudall WA (1997) Altered expression and activity of G1/S cyclins and cyclin-dependent kinases characterize squamous cell carcinomas of the head and neck. *Int J Cancer* **73**: 551–555

Pomerantz J, Schreiber Agus N, Liegeois NJ, Silverman A, Alland L, Chin L, Potes J, Chen K, Orlow I, Lee HW, Cordon Cardo C and Depinho RA (1998) The Ink4a tumor suppressor gene product, p19Arf, interacts with MDM2 and neutralizes MDM2's inhibition of p53. *Cell* **92**: 713–723

Quelle DE, Zindy F, Ashmun RA and Sherr CJ (1995) Alternative reading frames of the INK4a tumor suppressor gene encode two unrelated proteins capable of inducing cell cycle arrest. *Cell* **83**: 993–1000

Quelle DE Cheng M, Ashmun RA and Sherr CJ (1997) Cancer-associated mutations at the INK4a locus cancel cell cycle arrest by p16INK4a but not by the alternative reading frame protein p19ARF. *Proc Natl Acad Sci USA* **94**: 669–673

Reed AL, Califano J, Cairns P, Westra WH, Jones RM, Koch W, Ahrendt S, Eby Y, Sewell D, Nawroz H, Bartek J and Sidransky D (1996) High frequency of p16 (CDKN2/MTS-1/INK4A) inactivation in head and neck squamous cell carcinoma. *Cancer Res* **56**: 3630–3633

Ruas M and Peters G (1998) The p16/INK4a/CDKN2A tumor suppressor and its relatives. *Biochim Biophys Acta* **1378**: 115–177

Serrano M, Hannon GJ and Beach D (1993) A new regulatory motif in cell-cycle control causing specific inhibition of cyclin D/CDK4 [see comments]. *Nature* **366**: 704–707

Serrano M, Lee H, Chin L, Cordon-Cardo C, Beach D and Depinho RA (1996) Role of the INK4a locus in tumour suppression and cell mortality. *Cell* **85**: 27–37

Shapiro GI, Park JE, Edwards CD, Mao L, Merlo A, Sidransky D, Ewen ME and Rollins BJ (1995) Multiple mechanisms of p16INK4A inactivation in non-small cell lung cancer cell lines. *Cancer Res* **55**: 6200–6209

Sherr CJ (1994) G1 phase progression: cycling on cue [see comments]. *Cell* **79**: 551–555

Smith CJ and Pindborg JJ (1969) *Histological Grading of Oral Epithelial Atypia by the Use of Photographic Standards*. C Hamburgers Bogtrykkeri: Copenhagen

Steingrimsdottir H, Penhallow J, Farzeneh F, Johnson N and Tavassoli M (1997) Detection of p53 mutations in oral cancer samples using a sensitive PCR-based method. *DNA Damage Mutagenesis* **25**: 315–318

Stone S, Jiang P, Dayananth P, Tavtigian SV, Katcher H, Parry D, Peters G and Kamb A (1995) Complex structure and regulation of the P16 (MTS1) locus. *Cancer Res* **55**: 2988–2994

Suzuki T, Kitao S, Matsushime H and Yoshida M (1996) HTLV-1 Tax protein interacts with cyclin-dependent kinase inhibitor p16INK4A and counteracts its inhibitory activity towards CDK4. *EMBO J* **15**: 1607–1614

Tam SW, Shay JW and Pagano M (1994) Differential expression and cell cycle regulation of the cyclin-dependent kinase 4 inhibitor p16Ink4. *Cancer Res* **54**: 5816–5820

Timmermann S, Hinds PW and Munger K (1997) Elevated activity of cyclin-dependent kinase 6 in human squamous cell carcinoma lines. *Cell Growth Differ* **8**: 361–370

Towbin H, Staehelin T and Gordon J (1992) Electrophoretic transfer of proteins from polyacrylamide gels to nitrocellulose sheets: procedure and some applications. 1979 [classical article]. *Biotechnology* **24**: 145–149

Van Der Riet P, Nawroz H, Hruban RH, Corio R, Tokino K, Koch W and Sidransky D (1994) Frequent loss of chromosome 9p21–22 early in head and neck cancer progression. *Cancer Res* **54**: 1156–1158

Wang HG, Draetta G and Moran E (1991) E1A induces phosphorylation of the retinoblastoma protein independently of direct physical association between the E1A and retinoblastoma products. *Mol Cell Biol* **11**: 4253–4265

Weinberg RA (1995) The retinoblastoma protein and cell cycle control. *Cell* **81**: 323–330

Welcker M, Lukas J, Strauss M and Bartek J (1996) Enhanced protein stability: a novel mechanism of D-type cyclin over-abundance identified in human sarcoma cells. *Oncogene* **13**: 419–425

Wong DT, Todd R, Tsuji T and Donoff RB (1996) Molecular biology of human oral cancer. *Crit Rev Oral Biol Med* **7**: 319–328

Xiao ZX, Chen J, Levine AJ, Modjtahedi N, Xing J, Sellers WR and Livingston DM (1995) Interaction between the retinoblastoma protein and the oncoprotein MDM2. *Nature* **375**: 694–697

Xiong Y, Hannon GJ, Zhang H, Casso D, Kobayashi R and Beach D (1993) p21 is a universal inhibitor of cyclin kinases [see comments]. *Nature* **366**: 701–704

Yeudall WA, Paterson IC, Patel V and Prime SS (1995) Presence of human papillomavirus sequences in tumour-derived human oral keratinocytes expressing mutant p53. *Eur J Cancer B Oral Oncol* **31B**: 136–143

Yoo GH, Xu HJ, Brennan JA, Westra W, Hruban RH, Koch W, Benedict WF and Sidransky D (1994) Infrequent inactivation of the retinoblastoma gene despite frequent loss of chromosome 13q in head and neck squamous cell carcinoma. *Cancer Res* **54**: 4603–4606

Yoshida S, Todoroki T, Ichikawa Y, Hanai S, Suzuki H, Hori M, Fukao K, Miwa M and Uchida K (1995) Mutations of p16Ink4/CDKN2 and p15Ink4B/MTS2 genes in biliary tract cancers. *Cancer Res* **55**: 2756–2760

Zhang SY, Klein Szanto AJ, Sauter ER, Shafarenko M, Mitsunaga S, Nobori T, Carson DA, Ridge JA and Goodrow TL (1994) Higher frequency of alterations in the p16/CDKN2 gene in squamous cell carcinoma cell lines than in primary tumors of the head and neck. *Cancer Res* **54**: 5050–5053

Zhang Y, Xiong Y and Yarbrough WG (1998) ARF promotes MDM2 degradation and stabilizes p53: ARF-INK4a locus deletion impairs both the Rb and p53 tumor suppression pathways. *Cell* **92**: 725–734

Zhou P, Jiang W, Weghorst CM and Weinstein IB (1996) Overexpression of cyclin D1 enhances gene amplification. *Cancer Res* **56**: 36–39

Zhou X, Tarmin L, Yin J, Jiang HY, Suzuki H, Rhyu MG, Abraham JM and Meltzer SJ (1994) The MTS1 gene is frequently mutated in primary human esophageal tumors. *Oncogene* **9**: 3737–3741

 *© Cancer Research Campaign 1999*
